# Selective Hyper-responsiveness of the Interferon System in Major Depressive Disorders and Depression Induced by Interferon Therapy

**DOI:** 10.1371/journal.pone.0038668

**Published:** 2012-06-06

**Authors:** Joerg F. Schlaak, Martin Trippler, Carolina Hoyo-Becerra, Yesim Erim, Bernhard Kis, Bo Wang, Norbert Scherbaum, Guido Gerken

**Affiliations:** 1 Department of Gastroenterology and Hepatology, University Hospital of Essen, Essen, Germany; 2 Department of Psychosomatics, Rheinische Kliniken, Essen, Germany; 3 Department of Psychiatry and Psychotherapy, LVR-Klinikum, Essen, Germany; 4 Addiction Research Group at the Department of Psychiatry and Psychotherapy, LVR-Klinikum, Essen, Germany; Kanazawa University, Japan

## Abstract

**Background:**

Though an important percentage of patients with chronic hepatitis C virus (HCV) undergoing interferon (IFN) therapy develop depressive symptoms, the role of the IFN system in the pathogenesis of depressive disorders is not well understood.

**Methods:**

50 patients with HCV infection were treated with standard combination therapy (pegylated IFN-α2a/ribavirin). IFN-induced gene expression was analyzed to identify genes which are differentially regulated in patients with or without IFN-induced depression. For validation, PBMC from 22 psychiatric patients with a severe depressive episode (SDE) and 11 controls were cultivated *in vitro* with pegylated IFN-α2a and gene expression was analyzed.

**Results:**

IFN-induced depression in HCV patients was associated with selective upregulation of 15 genes, including 6 genes that were previously described to be relevant for major depressive disorders or neuronal development. In addition, increased endogenous IFN-production and selective hyper-responsiveness of these genes to IFN stimulation were observed in SDE patients.

**Conclusions:**

Our data suggest that selective hyper-responsiveness to exogenous (IFN therapy) or endogenous (depressive disorders) type I IFNs may lead to the development of depressive symptoms. These data could lead to the discovery of novel therapeutic approaches to treat IFN-induced and major depressive disorders.

## Introduction

Chronic hepatitis C virus (HCV) infection is a major cause for liver-related morbidity and mortality affecting approximately 170 million individuals worldwide. Around 70% of patients develop histological evidence of chronic liver disease, which may ultimately lead to liver cirrhosis and hepatocellular carcinoma [1]. In recent studies using pegylated interferon (IFN)-α2 in combination with ribavirin response rates for genotype 1 patients were approximately 40–50% while patients with genotype 2 or 3 respond in about 80–90% [2]–[4]. A major obstacle for this combination therapy, however, is the development of depressive side effects that is observed in 22–31% of patients [2]–[4].

There is an increasing body of evidence that point at depression as an inflammatory disorder prompted by deregulated levels of pro-inflammatory cytokines, such interleukin-1β, IL-6, tumor necrosis factor-α, IFN-γ and IFN-γ-induced protein 10 (IP-10) [5]–[9]. It is known that IFN-α is a pro-inflammatory innate immune cytokine that causes high rates of depression in humans. The IFN-α mediated depression has been widely reported in patients treated for hepatitis B, hepatitis C and malignant melanoma and can induce to suicidal behavior [7], [8], [10], [11]. Despite the increasing significance of this phenomenon, the molecular interactions underlying this outcome are poorly understood. Multiple mechanisms including immune, neurotransmitter and neuroendocrine pathways have been associated with IFN-α mediated depression, and a wide range of putative risk factors are being proposed and studied at genetic, molecular and behavioral levels. As cytokines affect the synthesis, release and cellular reuptake of monoamines and their dysregulation affects several central nervous system functions, it has been postulated that some neurophysiologic changes that affect activity of the noradrenergic and/or the serotonergic neuron system may occur during IFN therapy [12]–[14]. It has been shown that IFN-α can promote the depletion of serotonin by inducing the activation of indolamine 2,3 dioxygenase (*IDO1*), responsible of the conversion of tryptophan to kynurenine (12, 14, 15), and plays a crucial role as molecular mediator of inflammation-induced depressive-like behavior in mice [15]–[17]. Concomitantly, IFN-α may alter dopamine metabolism by changes in kynurenic acid, a tryptophan metabolite that can affect dopamine release, and decreased concentrations of tetrahydrobiopterin (BH4) as an enzyme cofactor for tyrosine hydroxylase, which is the rate limiting enzyme in the synthesis of dopamine (13, 15). Moreover, it has been shown that IFN-α is a potent inducer of mitogen activated protein kinase (MAPK) signaling pathways [18], that have been reported to activate monoamine transporters and thereby deplete their synaptic concentrations [13], [19]. Studies in experimental animals have revealed that IFN-α, when injected into the ventricular system, can suppress serotonin (5-HT) and dopamine levels in the striatum, frontal cortex and midbrain [12], [14]. IFN-α may also indirectly stimulate the hypothalamic-pituitary-adrenal axis, which is known to be overactive in depression, by inducing cytokines (e.g. IL-6), that activate this system [20]–[22]. Interestingly, administration of IFN-α to non-human primates has been associated with immune, neuroendocrine and behavioral responses similar to that observed in humans. Thus, it has been found to increase plasma adrenocorticotrophic hormone, cortisol and IL-6, to decrease corticotrophin-releasing factor and to promote a depressive-like, huddling behavior [23]. Concomitantly, most of these molecular alterations are represented by genetic signatures, which have been reported in a variety of studies relating the prevalence of genetic polymorphisms on monoamine- and interleukin- related pathways with the development of IFN-induced depression (i.e., *IDO1*, serotonin receptor and transporter polymorphisms [24]–[27], and IL-6 polymorphisms [24], reviewed in [28]). Some of them are widely studied in case of idiopathic depression as well (reviewed in [29]). The growing evidences about the role of exogenous IFN on depression have promoted that genetic surveys extend towards the study of the IFN-related pathways. Thus, polymorphisms in IFN receptor alpha 1 have been proposed as a risk factor for the development of depressive symptoms during IFN-α therapy [30]. Further studies in mice have suggested that systemic IFN-α treatment may also have a direct effect by upregulating IFN-stimulated genes (ISGs) in the brain with a profile very similar to peripheral organs [31]. Until this point it was not clear, however, which ISGs are directly or indirectly involved in the induction of depression.

Therefore, we have studied *in vivo* the primary transcriptional response to IFN-α in patients treated for chronic hepatitis C to identify target genes that mediate the depressive side effects of IFN-α. With the aim to conjugate both, idiopathic- and IFN-derived-depression genetic background, the results were further validated *in vitro* in psychiatric patients with a severe depressive episode.

## Materials and Methods

### Patients

A total of 50 treatment naïve patients with chronic hepatitis C that fulfilled standard inclusion criteria as described previously [3] were included into this single-site prospective study (Table 1). They were treated with pegylated IFN-α2a (Pegasys, Roche; 180 µg s.c. once weekly) in combination with oral ribavirin (body weight < 75 kg: 1000 mg/d; body weight > 75 kg: 1200 mg/d) for 12 months (HCV genotype 1/4, n = 41/1) or 6 months (HCV genotype 2/3, n = 1/7), respectively.

**Table 1:** Characteristics of the study participants.

	HCV (n = 50)	SDE (n = 22)	Control (n = 11)
**Age [years], mean ± SEM**	43.62±15.75	51.23±38.60	36.22±23.22
**Female, n (%)**	20 (40)	14 (60,9)	6 (54.6)
**BMI [kg/m^2^], mean ± SEM**	25.59±2.51	25.37±3.87	23.42±3.68
**ALT [U/L], mean ± SEM**	97.22±145.45	25.52±40.05	–
**HCV genotype 1, n (%)**	41 (82)	–	–
**HCV load [IU/mL], mean ± SEM**	1.16 E+06±2.58 E+11	–	–
**SVR, drop, n (%)**	19 (38) / 4 (8)	–	–
**Inflammation grade 0,1,2,3,4, n.d., n (%)**	2 (4), 22 (44), 17 (34), 1 (2), 1 (2) ,7 (14)	–	–
**Fibrosis stage 0,1,2,3,4, n.d., n (%)**	6 (12), 15 (30), 11 (22), 7 (14), 4 (8), 7 (14)	–	–
**De novo depression, n (%)**	11(22), 39 (78)	–	–
**Pre-existing psychiatric disorder, n (%)**	9 (18)	–	–
**Psychotropic drugs use, n (%)**	8 (16)	–	–
**Pre-existing heroin abuse, n (%)**	4 (8)	–	–
**Heroin abuse, n (%)**	2 (4)	–	–
**Acute SDE, n (%)**	–	2 (6.7)	–
**RDD + acute SDE, n (%)**	–	18 (78.3)	–
**RDD + acute SDE + psych. symptoms, n (%)**	–	1 (4.4)	–
**Bipolar disorder + acute SDE, n (%)**	–	3 (13.0)	–
**HAMD-17, mean ± SEM**	–	23.11±3.15	–

Abbreviations: HCV  =  hepatitis C virus, BMI  =  body mass index, ALT  =  Alanine aminotransferase, SVR  =  sustained viral response, drop  =  study drop-out, HAMD =  Hamilton Depression Rating Scale, Psych.  =  psychotic, RDD  =  recurrent depressive disorder, SDE  =  severe depressive episode, SEM  =  standard error of the mean, n.d.  =  not determined.

22 psychiatric patients hospitalized for a severe depressive episode (SDE) were prospectively analyzed to validate the target genes in an independent cohort. The diagnosis of a SDE was based on the psychiatric evaluation by a board-certified psychiatrist. The severity of depression was assessed using the 17-item Hamilton Depression Rating Scale (HAMD-17) that is a common tool in clinical trials [32]. A recurrent depressive disorder was diagnosed in 17 patients (ICD-10: F33.2), one of them with psychotic symptoms (ICD-10: F33.3), 3 patients had a bipolar affective disorder with a severe depressive episode (ICD-10: F31.4), and two patients a severe depressive episode (ICD-10: F32.2).

All parts of study were approved by the local ethical committee at the University Hospital of Essen. Patients from both patient cohorts and relatives of the deceased gave their written informed consent.

### Diagnosis of Depression During IFN Therapy

Before IFN therapy all HCV patients filled out questionnaires (Hospital Anxiety and Depression Scale (HADS) and Beck Depression Inventory (BDI)) to quantify possible preexisting depressive symptoms. Depression scores were measured three, six and twelve months after IFN therapy. The structured psychiatric interview Mini-DIPS was conducted in 47 patients with known or suspected pre-existing psychiatric disorders by a board-certified psychiatrist resulting in the diagnosis of previous intravenous heroin abuse (n = 8) and several psychiatric disorders (Table 1) in 15 of the patients. Patients with schizophrenia were treated with neuroleptic medication before and during IFN therapy. Three patients received antidepressive medication before initiation of therapy. Eleven patients developed moderate *de novo* depressive symptoms and were treated with antidepressive medication of citalopram 20 mg per day. Symptoms of depression improved under this therapy and no patient discontinued IFN therapy prematurely.

### Isolation and *in vitro* Stimulation of PBMC

Blood samples were taken from healthy controls (n = 11) or patients hospitalized for a severe depressive episode (n = 22). Then, PBMC were isolated as previously described [33] and cultured for 16 h in the absence or presence of 100 U/mL pegylated IFN-α2a followed by isolation of total RNA as described later. All experiments were performed in triplicate under stringent endotoxin-free conditions.

### Isolation of Total RNA from Peripheral Blood, Cultivated PBMC

For *in vivo* gene expression analysis, peripheral blood was collected directly into PAXgene Blood RNA Tubes (Becton Dickinson, Heidelberg, Germany) and RNA was isolated using the PAXgene Blood RNA Kit (Qiagen, Hilden, Germany) according to the instructions of the manufacturer.

For *in vitro* gene expression analysis, total RNA was isolated from cultivated PBMC using Trizol (Invitrogen, Karlsruhe, Germany) followed by a cleanup procedure using the RNeasy Mini Kit and the RNase-Free DNase Set (both from Qiagen) following the manufacturer’s protocol.

### DNA Microarray Analysis

Double-stranded cDNA was synthesized from 20 µg of total RNA using Superscript II (Gibco, Gaithersburg, MD) and an oligo T-7-(dT)24 primer. cRNA was synthesized using a primer that contained a T-7 RNA polymerase site that is labeled with biotin-11-CTP and biotin-16-UTP using a BioArray T-7 polymerase labeling kit (Enzo, Farmingdale, NY) following the manufacturer’s protocol. Hybridizing, washing, antibody amplification, and staining of probe arrays are performed according to the instructions of the manufacturer. Experiments were performed using human genomic microarrays (HG-U133A 2.0, Affymetrix, Santa Clara, CA). GeneChip Operating Software (GCOS v1.2.0.037, Affymetrix) was used to perform absolute analyses of individual microarrays followed by comparison analyses between individual off- an on-treatment samples. Data filtering was performed using the Data Mining Tool software (DMT v3.1, Affymetrix).

### Real-time Detection Gene Expression with One-step RT-PCR

In order to determine the gene expression levels of the candidate ISGs, endogenous IFNs and TLRs one-step RT-PCR with real-time detection was performed on the Rotor-Gene 2000 real-time amplification system (Corbett Research, Mortlake, Australia). One-step RT-PCR was carried out with the QuantiTect SYBR Green RT-PCR Kit (Qiagen) according to the manufacturer’s instructions as described before [34]. Three house-keeping genes β-actin (*ACTB)*, tyrosine 3-monooxygenase/tryptophan 5-monooxygenase activation protein (YWHAZ), and β2-microglobulin (B2M) were quantified for normalization of gene copy numbers to the variable RNA amounts within the different samples. For each gene data are shown as copy numbers normalized to the number of *ACTB* transcripts in the sample.

Self-designed primers were used for *ACTB*, *B2M*, *GBP1*, *IFIT1*, *ISG15*, *MX1*, *STAT1*, and *YWHAZ* (Table 2). For all other genes commercial primers were used (QuantiTect Primer Assay, Qiagen).

**Table 2:** Self-designed primers used for quantitative real time RT-PCR.

Gene	Accession Number	Forward Primer (5′−3′)	Reverse Primer (5′−3′)
*ACTB*	BC016045	TCCCTGGAGAAGAGCTACGA	AGCACTGTGTTGGCGTACAG
*B2M*	NM_004048	CAAATTCTGCTTGCTTGCTTT	TGGAGCAACCTGCTCAGATAC
*GBP1*	NM_002053	TTGCTGAAAGAGCAAGAGAGG	TGGTTAGGGGTGACAGGAAG
*IFIT1*	NM_001548	GCCCAGACTTACCTGGACAA	GGTTTTCAGGGTCCACTTCA
*ISG15*	NM_005101	TGTCGGTGTCAGAGCTGAAG	AGAGGTTCGTCGCATTTGTC
*MX1*	NM_002462	AGCCACTGGACTGACGACTT	GAGGGCTGAAAATCCCTTTC
*STAT1*	NM_007315	CCGTTTTCATGACCTCCTGT	TGAATATTCCCCGACTGAGC
*YWHAZ*	NM_145690	ATCCATGCTGTCCCACAAA	TGGCCACCTCAAGATGAAA

Abbreviations: RT-PCR  =  reverse transcription polymerase chain reaction.

### Statistical Methods

Prior to significance and prediction analyses the different chip raw data were normalized by means of background adjustment and quantile normalization using the RMAExpress v0.4.1 software [35]. To identify genes which are differentially expressed in HCV patients with or without IFN induced depression the Significance Analysis of Microarrays (SAM v3.0) Excel add-in was used [36]. SAM computes repeated permutations of the data to determine if the expression of any genes are significantly related to a given response variable (i.e. before or after IFN treatment). The cut off for significance is determined by a tuning parameter delta, chosen to minimize the false positive rate.

Class prediction analysis was performed using the Prediction Analysis for Microarrays (PAM v2.1) Excel add-in to identify genes that best characterize each of two given classes (IFN induced depression or no depression) using the nearest shrunken centroid method [37].

Statistical analysis of the gene expression levels was performed using the T-Test or Mann-Whitney test as appropriate using the GraphPad Prism software (version 4.03). The null hypothesis was rejected at the p≤0.05 level.

## Results

As described previously, 22% (11/50) of the HCV patients treated with pegylated IFN-α2a and ribavirin developed depressive side effects during therapy [3]. Development of depressive side effects did not correlate with response to therapy, sex, age, ALT-levels, presence of cirrhosis, HCV genotype, viral load before therapy or response to therapy (data not shown).

To identify candidate genes which mediate the depressive side effects of IFN-α, microarray analysis of the primary transcriptional response to IFN-α was performed in those 11 patients that developed depression during therapy in comparison to 11 randomly chosen HCV patients that did not experience such side effects. Using significance and class prediction analysis, a total of 15 genes were identified that were selectively hyper-responsive to exogenous IFN-α in patients that developed depressive side effects (Table 3). In addition, considering the previous reported association with IFN-related depression [13], [18], [19], [38], the microarray results of *IP-10* and *IDO1* underwent statistical analysis. Although a trend towards an increased expression was observed, changes were not statistically significant which may be due to the small sample population and the very early time point (12h) studied after IFN injection (data not shown).

**Table 3:** Genes associated with interferon-induced depression.

Gene	Full gene name / functional association	Transcript ID
*DISC1*	disrupted in schizophrenia 1 / alterations of hippocampal structure and function, neurite outgrowthand cortical development, neuron migration, neuroblast proliferation	NM_018662
*DYNLT1*	dynein, light chain, Tctex-type 1 / hippocampal neuron development (e.g. neurite sprouting, axonspecification, dendritic elaboration)	NM_006519
*GBP1*	guanylate binding protein 1, interferon-inducible / ISG	NM_002053
*GCH1*	GTP cyclohydrolase 1 / bipolar disorder, depression, anxiety, dopamine biosynthesis	NM_000161
*GLRX*	glutaredoxin (thioltransferase) / ISG	NM_002064
*MEF2A*	MADS box transcription enhancer factor 2, polypeptide A (myocyte enhancer factor 2A) / neuronaldifferentiation, suppression of hippocampal excitatory synapse number, postsynaptic differentiation	NM_005587
*PSMB9*	proteasome (prosome, macropain) subunit, beta type, 9 (large multifunctional peptidase 2) / ISG	NM_002800
*RBCK1*	RanBP-type and C3HC4-type zinc finger containing 1 / ISG	NM_006462
*RTP4*	receptor (chemosensory) transporter protein 4 / ISG	NM_022147
*ST3GAL5*	ST3 beta-galactoside alpha-2,3-sialyltransferase 5 / apoptosis in mouse hippocampal cell lines;Amish infantile epilepsy syndrome; ganglioside biosynthesis	NM_003896
*STAT1*	signal transducer and activator of transcription 1 / ISG	NM_007315
*TNFSF10*	tumor necrosis factor (ligand) superfamily, member 10 / ISG	NM_003810
*TOR1B*	torsin family 1, member B (torsin B) / idiopathic dystonia, recurrent major depression; widespreadneuronal expression; regulation of neurotransmitter release	NM_014506
*UBE2L6*	ubiquitin-conjugating enzyme E2L 6 / ISG	NM_004223
*ZNF200*	zinc finger protein 200 / ISG	NM_003454

Abbreviations: ISG  =  interferon stimulated gene.

**Figure 1:**
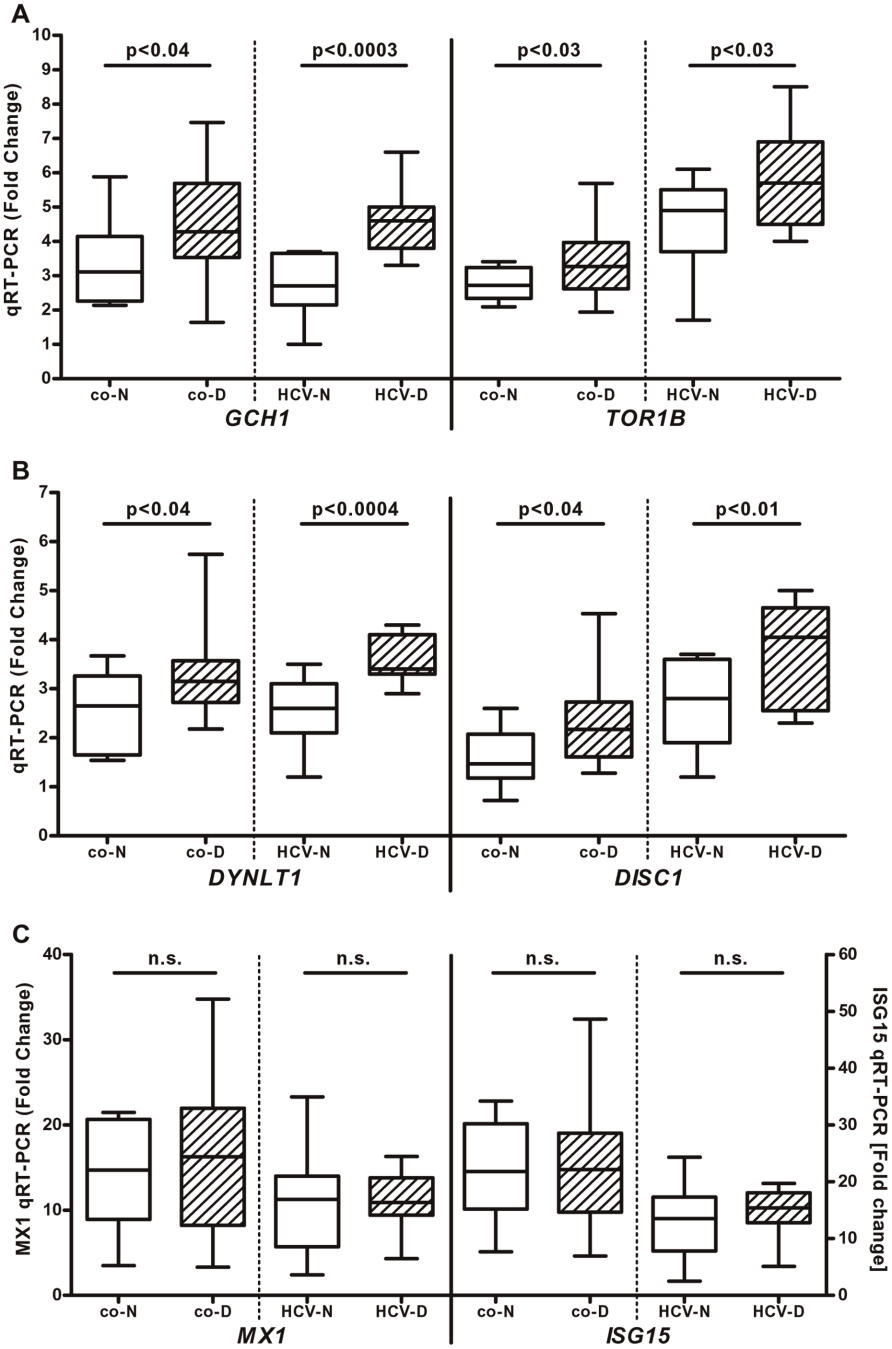
Enhanced IFN-mediated induction of selective ISGs in HCV patients with IFN-induced depression (*in vivo*) and psychiatric patients with a severe depressive episode (SDE, *in vitro*). Total RNA was isolated from peripheral blood of hepatitis C virus (HCV) infected patients with (n = 11, “HCV-D”) or without (n = 11, “HCV-N”) IFN-induced depression 12 hours before and 12 hours after the first injection of pegylated IFN-α2a. Expression of IFN stimulated genes (ISGs) was analyzed by quantitative RT-PCR (panel A: *GCH1*, *TOR1B*; panel B: *DYNLT1*, *DISC1*; panel C: *MX1*, *ISG15*). To validate the data in an independent cohort, PBMC were isolated from 11 healthy controls (“co-N”) and 22 patients hospitalized for a SDE (“co-D”) and stimulated with 100 U/mL pegylated IFN-α2a *in vitro* for 16 h followed by isolation of total RNA. Data are shown as box plots (range, 25% and 75% percentile, mean).

To validate these candidate genes in different patient populations under different experimental conditions, the *in vitro* response to pegylated IFN-α2a was studied in a cohort of 22 psychiatric patients that were hospitalized for a SDE. Compared to healthy controls, pegylated IFN-α2a led to a significantly higher induction of *GCH1*, *TOR1B* (Figure 1A), *DYNLT1* and *DISC1* (Figure 1B) while there was a trend towards higher induction for *MEF2A* and *ST3GAL5* (data not shown). No difference was observed for classical ISGs like *MX1* or *ISG15* (Figure 1C) as well as *IFIT1* and *IFI16* (data not shown) suggesting that there is a selective rather than a general hyper-responsiveness to type I IFNs in these patients.

**Figure 2:**
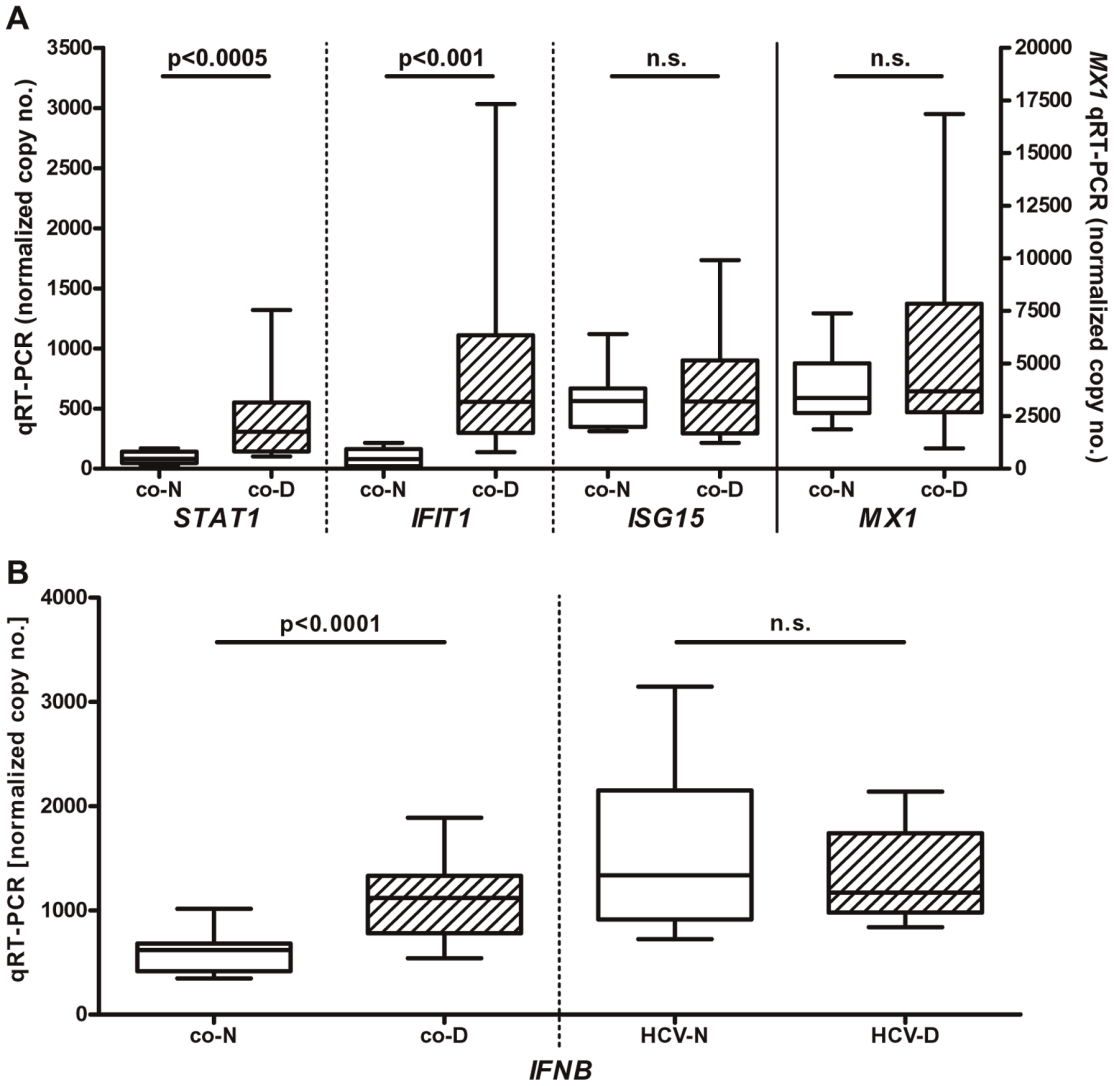
Enhanced ISG expression and IFN-production in psychiatric patients with a severe depressive episode (SDE). Panel A. After 24 h of *in vitro* incubation without any further stimuli, total RNA was isolated from peripheral blood mononuclear cells of 11 healthy controls (“co-N”) and 22 patients hospitalized for a SDE (“co-D”). Panel B. Total RNA was isolated directly from unseparated peripheral blood of healthy controls (“co-N”, n = 11), SDE patients (“co-D, n = 22”) and HCV patients without (“HCV-N”, n = 11) or with (“HCV-D”, n = 11) IFN-induced depression. Expression of IFN stimulated genes (ISGs) and IFN-β was analyzed by quantitative RT-PCR. Data (copies per 100,000 copies of *ACTB*) are shown as box plots (range, 25% and 75% percentile, mean).

When baseline levels of ISGs were studied in patients with SDE after 24h without additional stimulation, a significant upregulation of classical ISGs (i.e. *STAT1*, *IFIT1*) compared to healthy controls was observed (Figure 2A) suggesting that there is an increased production of endogenous IFNs in these patients. To test this hypothesis, we assessed the *in vivo* baseline levels of the most abundant IFN-α subtypes (IFN-α1 and -α2), IFN-β and IFN-γ by quantitative RT-PCR (Table 4, Figure 2B). The most striking finding was a significant upregulation of IFN-β production in patients with SDE compared to controls. Also, there were increased mRNA levels of IFN-α1 and IFN-α2 and a trend towards higher levels of IFN-γ suggesting a broad activation of type I and II IFN production in SDE patients. Interestingly, IFN-β expression was profoundly enhanced in HCV patients before therapy consistent with a direct stimulation by HCV through the Toll-like receptor (TLR) system (Table 4). IFN-γ mRNA levels were elevated in HCV patients that could be explained by activation of the PBMC-derived immunity while no significant changes in IFN-α mRNA levels were observed.

**Table 4:** Basal expression of interferons and toll-like receptor genes in individuals with or without depressive disorders.

Gene	Peripheral blood^1^		
	Controls (n = 11)	SDE-P (n = 22)	t-test
	mean ± SEM	mean ± SEM	p value
*IFNA1*	5,026±530.5	6,363±357.9	0.04
*IFNA2*	274.9±24.5	352.9±24.8	0.05
*IFNB1*	585.0±63.0	1,113±79.5	0.0001
*IFNG*	26.5±4.7	59.5±16.3	n.s.
*TLR3*	144.8±23.5	112.8±16.1	n.s.
*TLR7*	659.2±58.89	690.1±124.6	n.s.
*TLR8*	<40.0±4.0	<40.04.0	n.d.

1 Data are shown as copies per 100,000 copies of *ACTB*.

Abbreviations: SDE-P  =  severe depression episode patients, SEM  =  standard error of the mean, n.s.  =  not significant, n.d.  =  not determined.

Type I and II IFNs were significantly upregulated in depressive individuals confirming the results from the peripheral blood of SDE patients (Table 4). Interestingly, we also found an upregulation of *TLR3* and *TLR7* that function as sensors of the innate immune system for viral structures in particular, reviewed in [39]. In HCV-positive individuals a significant upregulation of IFN-β was observed (p<0.005) compared to HCV-negative individuals.

## Discussion

Determining the role of genetic vulnerability to harmful side effects is becoming a crucial issue in order to progress toward individualized drug therapy with successful outcome. A major obstacle of IFN therapy for chronic hepatitis C is that about a quarter of these patients will develop depressive side effects that can even lead to suicide in some cases [2], [3]. The pathophysiology of IFN-induced depression, however, is not well understood. Most of the molecular dysregulations observed in IFN-related depression are similar to those described for idiopathic depression, suggesting the existence of common pathways to both disorders. In fact, the monocyte-T-lymphocyte hypothesis of mayor depression predicts an immune system activation subjacent to the pathophysiology of major depressive disorder [40], and the association of the depressive symptoms with an increased production of pro-inflammatory cytokines [5]–[8] may indicate that they are the result of a maladaptive response to immune activation. Pegylated IFN-α and ribavirin therapy, as source of an artificial pro-inflammatory cytokine, has been related to the risk of develop depressive behavior by affecting the monoamine and cytokine balance, but a common genetic background with idiopathic depression is not yet elucidated. Our data suggest that the development of depressive side effects during therapy with pegylated IFN-α and ribavirin for chronic hepatitis C is associated with a selective hyper-responsiveness of the IFN system. This leads to the disproportional upregulation of 15 genes including 6 genes that were previously reported to be relevant for recurrent major depression or neuronal development in the brain. The relevance of these genes was validated in a separate cohort of psychiatric patients hospitalized for a severe depressive episode by analyzing the *in vitro* induction of these ISGs by pegylated IFN-α. In these cohorts, we were also able to demonstrate enhanced basal production of endogenous IFNs, which may contribute to the cytokine levels dysregulation. The 15 target genes include *DYNLT1*, *GCH1*, *TOR1B*, *DISC1*, *MEF2A* and *ST3GAL5* that to date were never related to an IFN-α regulation while all of them have been described in association with brain development or depression. *TOR1B*, which shows immunoreactivity in all subfields of the hippocampus [41], is homologue to *TOR1A* (alternative name: *DYT1*), is associated with early-onset recurrent major depression and is involved in the regulation of dopamine release [42]. *DYNLT1* (alternative name: *TCTEL1*) plays a key role in multiple steps of hippocampal neuron development such as neurite sprouting, axon specification and dendritic elaboration [43]. *MEF2A* is also involved in neuronal differentiation and postsynaptic differentiation [44]–[46]. *DISC1* plays a role in neurite outgrowth and cortical development and contributes to alterations of hippocampal structure and function [47], [48]. Furthermore, genetic analyses revealed an association with schizophrenia and major depression [49]–[52]. *ST3GAL5* is involved in neuronal apoptotic cell death in mouse hippocampal cell lines [53]. Finally, *GCH1* is the rate-limiting enzyme in BH4 biosynthesis, an essential cofactor required by the aromatic amino acid hydroxylase and nitric oxide synthase which are in turn the rate-limiting enzymes in dopamine and serotonin biosynthesis. Interestingly, it has already been shown that different IFN types, including pegylated-IFN forms, are able to induce *GCH1* activation with the subsequent increase of BH4 and its precursor neopterin, released from monocytes and macrophages [54], [55], which has been widely used as a pharmacodynamic marker in the evaluation and optimization of IFN therapy [56], [57]. Even more, several authors have lately reported that IFN-α therapy impairs phenylalanine metabolism in HCV infected individuals [58], [59], suggesting that behavioural side effects may be associated with the modulation of BH4 levels, thus affecting dopamine, serotonin and noradrenaline biochemistry. Clinically, alterations of *GCH1* activity has been associated with bipolar disorders, depression, anxiety, dystonia and deafness [60], [61]. The hippocampus is one of several limbic brain structures implicated in the pathophysiology and treatment of mood disorders. Recently, it has been suggested that depression may have a neurogenic origin as loss of neurons in the adult hippocampus is observed and neurogenesis is required for the actions of antidepressants [62], [63]. Furthermore, it has been demonstrated that exogenous administration of IFN-α suppressed neuronal proliferation via proinflammatory cytokines IL-1β and TNF-α in the hippocampus of adult rats [64]. Previous studies revealed that the exposition to these cytokines induce depressive symptoms in humans and depression-like behavior in animals. Latter findings even related specifically increased plasma levels of the mentioned cytokines to the cause of severe depressive symptoms in HCV patients [65]. Our data suggest that IFN-α may interfere with these processes through modulation of the target genes identified in this study.

Patients with chronic HCV infection often report fatigue, depressive mood, impaired cognitive functions, and reduced quality of life [66]–[68]. As these symptoms do not correlate with severity of liver disease, hepatic encephalopathy, or history of intravenous drug use [66], it has been speculated that HCV itself may cause these alterations. This is supported by the fact that HCV RNA is detectable in the brain [69] or in cerebrospinal fluid leading to the hypothesis that HCV may cross the blood brain barrier by infected monocytes which could result in secondary infection of microglial cells [70]. It is well known that HCV can activate the production of IFN-β through activation of the Toll-like receptor system [71], [72], which explains the strong upregulation of IFN-β in the peripheral blood of HCV patients in our study. Microarray studies have indicated that increased type I IFN production occurs also in the livers of HCV-infected chimpanzees and humans [73], [74]. Therefore, we hypothesize that this endogenous IFN production may, at least in part, explain the depressive and cognitive disorders that are frequently seen in these patients.

Only little is known about the relevance of the IFN system for depressive episodes in the course of affective disorders. In accordance with our mRNA data, it has been shown that plasma levels of IFN-γ are higher in patients with depression [75]. Though the role of type I IFNs is still not well understood, it has been reported that they may help sustain the chronic inflammatory response promoting the recruitment of inflammatory monocytes [76] which may be involved in the pathophysiology of the depressive symptoms. In this context, our findings may point to a direct implication of monocyte/macrophage activation by IFN-α with an alteration of the tryptophan metabolism and the subsequent neurotransmitter dysregulation through *GCH1* modulation.

According to our results, this study may have major implications regarding a) the pathophysiology of IFN-induced depression, b) the pathophysiology of depressive disorders and cognitive dysfunctions in HCV patients and c) the relevance of the IFN system for severe depressive episodes in psychiatric diseases. Still, possible limitations regarding the relative small sample size, the different medical treatment taken by some HCV and psychiatric patients or comparison of related but not identical psychiatric disorders must be considered. Our study design does not allow to clearly differentiate the alterations solely related to interferon therapy from another medication, drug or altered physiological condition. This would be possible to resolve by studying only patients without any previous psychiatric history and treatment, but such a group would be too difficult to obtain due to the demographic characteristics of HCV and psychiatric patients. Thus, the conclusions presented here need to be confirmed in independent larger cohorts. Nevertheless, this work provides valuable information about the possible mechanisms underlying depression and other cognitive dysfunctions affecting HCV patients treated with the current standard therapy and, as a last resort, giving the likelihood to try to prevent, mitigate or avoid such side effects. Collectively, our results extend across two completely different clinical scenarios to generate convergent data that bridge the phenomenon of IFN-induced depression and major depression and thereby provide a pathophysiologic mechanism of depressive disorders focused on the role of inflammatory cytokines.

In conclusion, these data suggest that selective hyper-responsiveness to exogenous or endogenous type I IFNs may lead to the development of depressive symptoms. This sheds new light on the pathogenesis of IFN-induced and depressive episodes and could ultimately lead to the discovery of novel therapeutic approaches to treat these conditions.
